# Network pharmacology of the regulation of Th17/Treg cell immune balance in the pathogenesis of arthritis by the combination of Chuanxiong Rhizoma, Radix Angelicae Biseratae and Achyranthis Bidentatae Radix

**DOI:** 10.1097/MD.0000000000042238

**Published:** 2025-04-25

**Authors:** Zhiqing Chen, Chaoyi Yin, Binshan Zhang, Zhen Li

**Affiliations:** a Dongguan Hospital of Guangzhou University of Chinese Medicine, Dongguan, Guangdong Province, China.

**Keywords:** arthritis, Chuanxiong Rhizoma, immune balance, network pharmacology, Radix Angelicae Biseratae and Achyranthis Bidentatae Radix combination, regulatory effect, Th17/Treg cells

## Abstract

This study explores the network pharmacological mechanism of the regulatory effect of Chuanxiong Rhizoma, Radix Angelicae Biseratae and Achyranthis Bidentatae Radix combination on Th17/Treg cell immune balance in the pathogenesis of arthritis. Databases such as the Chinese Pharmacopoeia, TCMSP, and BATMAN-TCM were used to obtain the active ingredients of Chuanxiong Rhizoma, Radix Angelicae Biseratae and Achyranthis Bidentatae Radix. After screening and weight removal, the target of action was determined using the TCMSP database. GeneCards and OMIN databases were used to screen the relevant targets for arthritis; A drug active ingredient–arthritis–target network model was constructed using Cytoscape and further analyze it; Protein–protein interaction networks were built using STRING database; R4.0.2 software was used to complete gene ontology analysis of intersection targets and enrichment of Kyoto Gene and Gene Encyclopedia pathway. After screening and weight removal, a total of 14 effective chemical components were obtained, corresponding to a total of 340 targets. After screening and weight removal, 171 active component targets were ultimately obtained. Transforming protein–proteins into gene names resulted in a total of 75 targets, which were used as candidate target sources for prescription. The drug targets of Chuanxiong Rhizoma, Radix Angelicae Biseratae and Achyranthis Bidentatae Radix, and arthritis were imported into R software, and 41 targets were obtained with relevant tools; Using the CytoNCA plug-in to complete the PPT further topology, PTGS2, AKT1, CASP3, CAT, and SOD1 are the top 5 nodes in terms of degree values; gene ontology enrichment analysis obtained 1332 entries, including 1120 biological process entries, 112 cell composition entries, and 100 molecular functions. Chuanxiong Rhizoma, Radix Angelicae Biseratae and Achyranthis Bidentatae Radix in combination can produce positive effects on the pathophysiology of arthritis and can enhance the synergistic effects of several medications. The immunological balance of Th17/Treg cells may have a regulatory role in the pathway.

## 1. Introduction

As the aging population gets older, the prevalence of arthritis is increasing.^[1]^ In 2020, there was a 95% rise in the global age-standardized rate of years lived with disability for total osteoarthritis compared to 1990.^[2]^ The continuous development of the disease not only consumes a lot of medical resources, but also leads to long-term physical and psychological pain, which affects the quality of life of patients and increases the social burden.^[3]^ Since there are now few clinically available medications for arthritis, patients’ symptoms can only be momentarily relieved by conventional therapies, which find it challenging to impede the underlying pathogenic process.^[4,5]^ Pang Ting et al’s research^[6]^ demonstrated that the body’s immunological equilibrium is crucial to the development of arthritis. Under normal conditions, T helper 17 cells with pro-inflammatory effects and T regulatory cells with anti-inflammatory effects are in dynamic balance in the body. However, the Th17/Treg balance is disrupted when there is a rise in Th17 cells or a fall in T cells, which helps to foster the onset of inflammatory responses and ultimately contributes to the development of arthritis.^[7,8]^ The working mechanism of Chinese medicines is intricate and challenging to pinpoint, particularly for Chinese medicine compounds that have several targets and effects of action. Guanjietong Pian is a hospital preparation from Dongguan Hospital of Traditional Chinese Medicine. With Chuanxiong Rhizoma, Radix Angelicae Biseratae and Achyranthis Bidentatae Radix combination as its monarch, Guanjietong Pian has the ability to tonify the liver and kidney, ease pain, promote qi and blood circulation, facilitate joints, and have good clinical efficacy in slowing the progression of osteoarthritis.^[9,10]^ Network pharmacology is an interdisciplinary subject based on the integration of informatics and biology, which can clarify the signaling pathways and molecular targets involved in drug treatment of diseases from multiple angles and levels through network analysis to study the mechanism of action of Chinese medicine prescriptions and screen active ingredients. Thus, in terms of Th17/Treg cell immunological homeostasis, the present study focuses on the network pharmacological mechanism of the regulating action of the Chuanxiong Rhizoma, Radix Angelicae Biseratae and Achyranthis Bidentatae Radix combination in the pathogenesis of arthritis.

## 2. Information and methods

### 2.1. Collection of disease targets

Literature on TCM drug intervention in the treatment of arthritis was screened in Chinese Pharmacopoeia, TCMSP, BATMAN-TCM and other databases. The following key words were used in Chinese and English: Chuanxiong Rhizoma, Radix Angelicae Biseratae and Achyranthis Bidentatae Radix combination; Arthritis; TH17 Treg cells; Immune balance. The search time for each database is from the establishment of the database until April 2023. The known targets in the pathophysiology of arthritis were found by searching and screening the known disease targets, and deduplicating and combining the discovered targets.^[11]^

### 2.2. Collection of Chinese herbal components and component targets

Identification of active chemical constituents of Chinese herbal medicines using TCM systems pharmacology platforms TCMSP (Chuanxiong Rhizoma, Radix Angelicae Biseratae, Achyranthis Bidentatae Radix), and the screening was completed based on the toxicokinetics of the constituents. The active components of Chuanxiong Rhizoma, Radix Angelicae Biseratae and Achyranthis Bidentatae Radix combination were identified by screening in TCMSP, with an oral bioavailability ≥30.0%, drug-likeness ≥0.18, and drug half-life ≥4 hours. Therefore, based on the combination characteristics and corresponding targets of Chuanxiong Rhizoma, Radix Angelicae Biseratae and Achyranthis Bidentatae Radix combination, the combination of drugs needs to be further screened. With the help of the NCBI Gene database, the target of TCM components acquired above was searched, the species “Homo sapiens” was chosen, the formal name was established, and pertinent data was added.^[12]^

### 2.3. Methodology for the construction of network pharmacology

(1) Target–protein interactions network: Using a Venn diagram, the common target information of arthritis targets and Chuanxiong Rhizoma, Radix Angelicae Biseratae and Achyranthis Bidentatae Radix combination was obtained. To build the protein–protein interaction (PPI) network model, the acquired common target data was loaded into the STRING database with the species set to human. To identify the important target proteins, the PPI network model was loaded into the Cytoscape 3.2.1 program and analyzed using the “Network Analysis” analysis. (2) Cytoscape network diagram construction: Following the aforementioned procedures, DAVID database and R4.0.2 software were used to complete the gene ontology (GO) and Kyoto Gene and Gene Encyclopedia (KEGG) enrichment analysis of the arthritis targets of the core drug combinations.^[13]^ The results of the GO and KEGG analyses were considered to be statistically different at *P* < .05. At the same time, Using the STRING database and Cytoscape, an Excel graph was created that included a summary of the active components, potential target proteins, and KEGG pathway annotations of the main medication combination of Chuanxiong Rhizoma, Radix Angelicae Biseratae and Achyranthis Bidentatae Radix combination for the treatment of arthritis. The network diagram, “Constructing drug active ingredient–arthritis–target network model,” was constructed, and the network topology analysis was completed.^[14]^

## 3. Results

### 3.1. Chemical composition and target analysis of Rhizoma Ligustici Chuanxiong Rhizoma, Radix Angelicae Biseratae and Achyranthis Bidentatae Radix combination

With oral bioavailability ≥30% and drug-likeness≥0.18 as the screening conditions, the components of Chuanxiong Rhizoma, Radix Angelicae Biseratae and Achyranthis Bidentatae Radix mentioned in the content determination of the Pharmacopoeia of Chinese Medicine that did not meet the screening conditions were also included in this study. After screening and weight reduction, a total of 14 effective chemical components were found, of which 4 were Chuanxiong Rhizoma, 7 were Radix Angelicae Biseratae, and 3 were Achyranthis Bidentatae Radix, equivalent to 340 targets overall. Screening and removal of duplicate targets, 171 active component candidates were ultimately obtained, as illustrated in Table 1.

**Table 1:** Chemical composition and target analysis of Chuanxiong Rhizoma, Radix Angelicae Biseratae and Achyranthis Bidentatae Radix concatenation.

Mol ID	PubChem ID	Title	OB/%	DL
MOL013287	442668	Betavulgarin	106.21	0.19
MOL013288	12315102	Rubrosterone	58.01	0.75
MOL000492	222284	Betasitosterol	54.83	0.24
MOL002055	5280343	Quercetin	47.07	0.28
MOL000098	10212	Ammidin	46.43	0.28
MOL002280	68081	Isoimperatorin	43.02	0.74
MOL002259	161409	Oacetylcolumbianetin	41.65	0.63
MOL000358	6313865	Angelol D	36.91	0.75
MOL000006	21669996	1R,2R,-2,3-dihydroxy-1-(7-methoxy-2-oxochromen-6-yl）-3-methylbutyl]-(Z）-2-methylbut-2-enoate	36.16	0.25
MOL013281	616303	Angelicone	35.83	0.21
MOL000608	21669996	[1R, 2R]-2,3-dihydroxy-1-(7-methoxy-2-oxochromen-6-yl）-3-methylbutyl]-(Z）-2-methylbut-2-enoate	81.41	0.51
MOL005823	73191	Nodakenin	61.67	0.52
MOL004328	10228	Osthole	59.29	0.21
MOL005100	6436246	Zosimin	47.74	0.27

DL = drug-likeness, OB = oral bioavailability.

### 3.2. Acquisition of arthritis targets and extraction of Chuanxiong Rhizoma, Radix Angelicae Biseratae and Achyranthis Bidentatae Radix combination common targets

**Figure 1.**
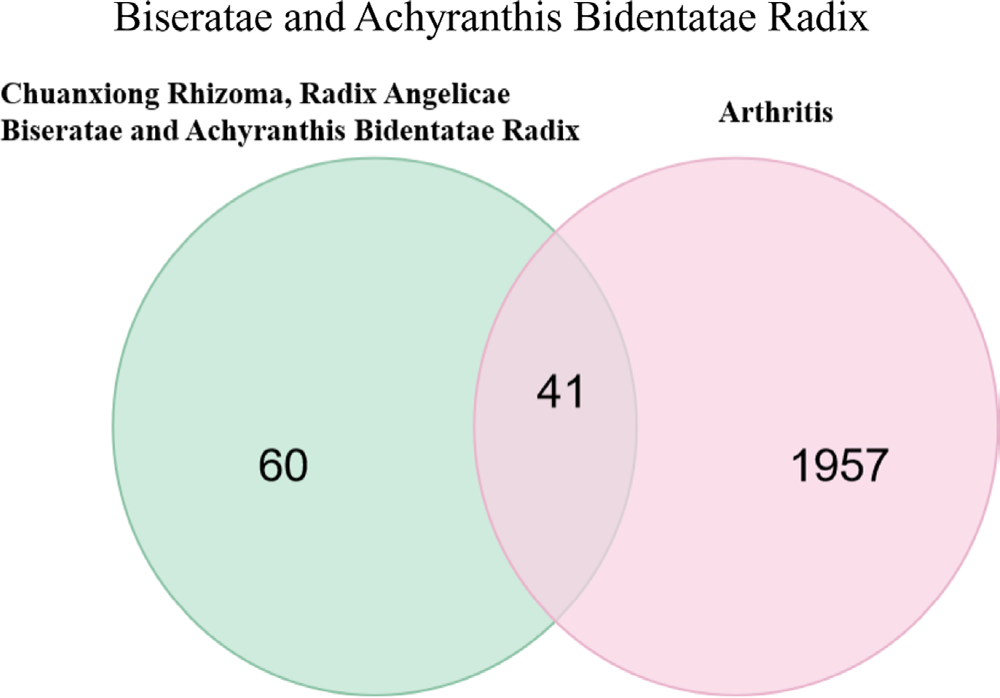
Wayne diagram of the common target of Chuanxiong Rhizoma, Radix Angelicae Biseratae and Achyranthis Bidentatae Radix.

Regular database searches were carried out, conditions were established in the Gene Cards database, and the basic targets from 2 databases were summarized and duplicate targets eliminated. After the protein–proteins were translated into gene names, 75 targets in all were found. These targets served as a source for potential targets for prescriptions. Hundred-one targets of Chuanxiong Rhizoma, Radix Angelicae Biseratae and Achyranthis Bidentatae Radix and 1998 targets of arthritis were obtained after deleting duplicates. After importing the arthritic and Chuanxiong Rhizoma, Radix Angelicae Biseratae and Achyranthis Bidentatae Radix medication targets into the R program, 41 targets were obtained with the use of relevant tools, as illustrated in Figure 1.

### 3.3. Modeling of drug active ingredient–arthritis–target network diagrams

**Figure 2.**
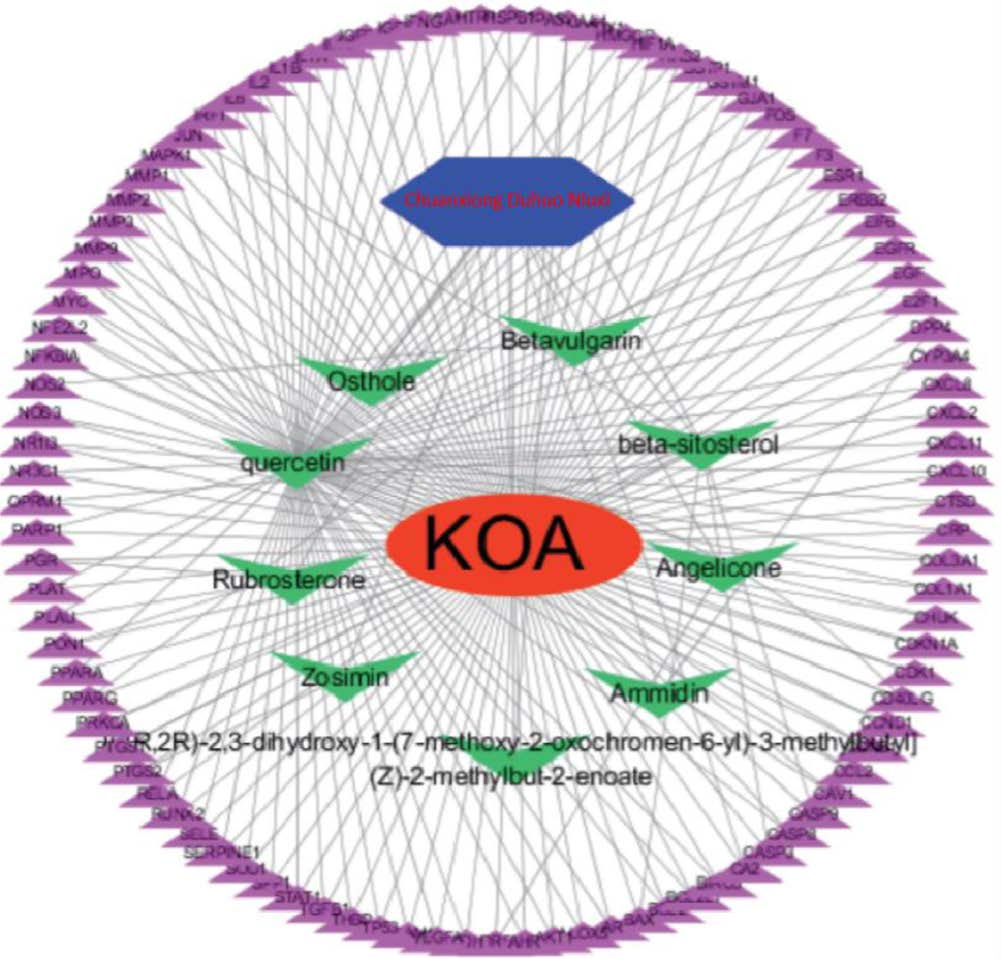
Network diagram of the drug active ingredient–arthritis–target network model (Note: The red core of the diagram is the Chuanxiong Rhizoma, Radix Angelicae Biseratae and Achyranthis Bidentatae Radix pairing. The surrounding green sections list the different components of the drug. Peripheral purple represents different component targets).

The active ingredient–arthritis–target network model was constructed using Cytoscape 3.7.1 with the active chemical components and common targets entered. The “Network Analysis” functional plug-in constructs the drug active ingredient–arthritis–target network diagram. It can be observed from the PPI diagram that the compounds connecting the target sites are osthole, betavulgarin, Betasitosterol, angelicone, ammidin, rubrosterone, quercetin, ammidin, and 1R，2R,-2，3-dihydroxy-1-(7-methoxy-2-oxochromen-6-yl）-3-methylbutyl]（Z）-2-methylbut-2-enoate. The Chuanxiong Rhizoma, Radix Angelicae Biseratae and Achyranthis Bidentatae Radix combination diagram was effective through multiple components and multiple targets. Refer to Figure 2.

### 3.4. Constructing the constituent–targets of the Chuanxiong Rhizoma, Radix Angelicae Biseratae and Achyranthis Bidentatae Radix

**Figure 3.**
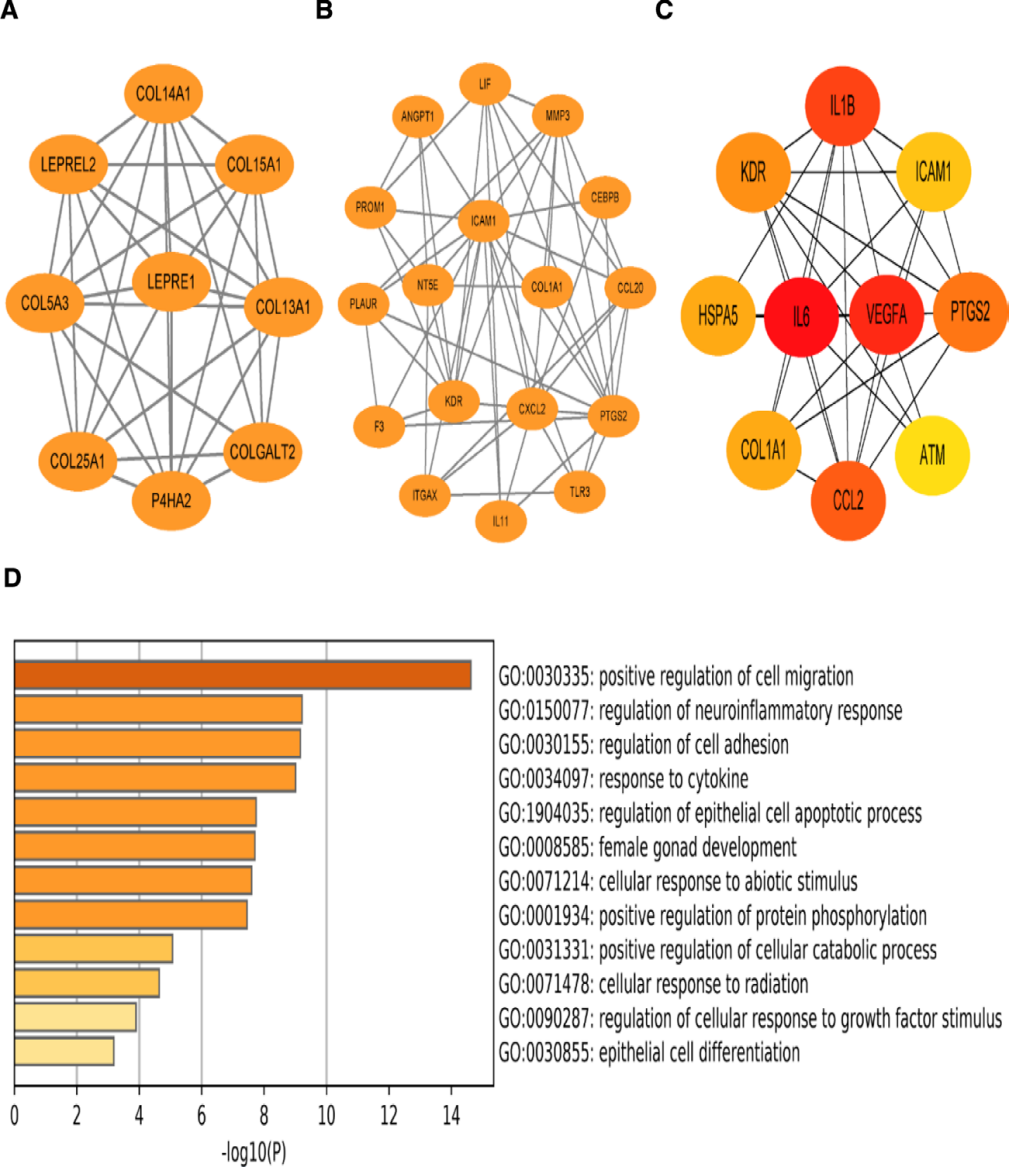
Key target of Chuanxiong Rhizoma, Radix Angelicae Biseratae and Achyranthis Bidentatae Radix combination (Note: Figures A, B, and C show the key nodes in the PPI network, demonstrating the connection between different targets; Figure D shows the relative expression content of genes in different targets, with the content decreasing sequentially from top to bottom). PPI = protein–protein interaction.

**Figure 4.**
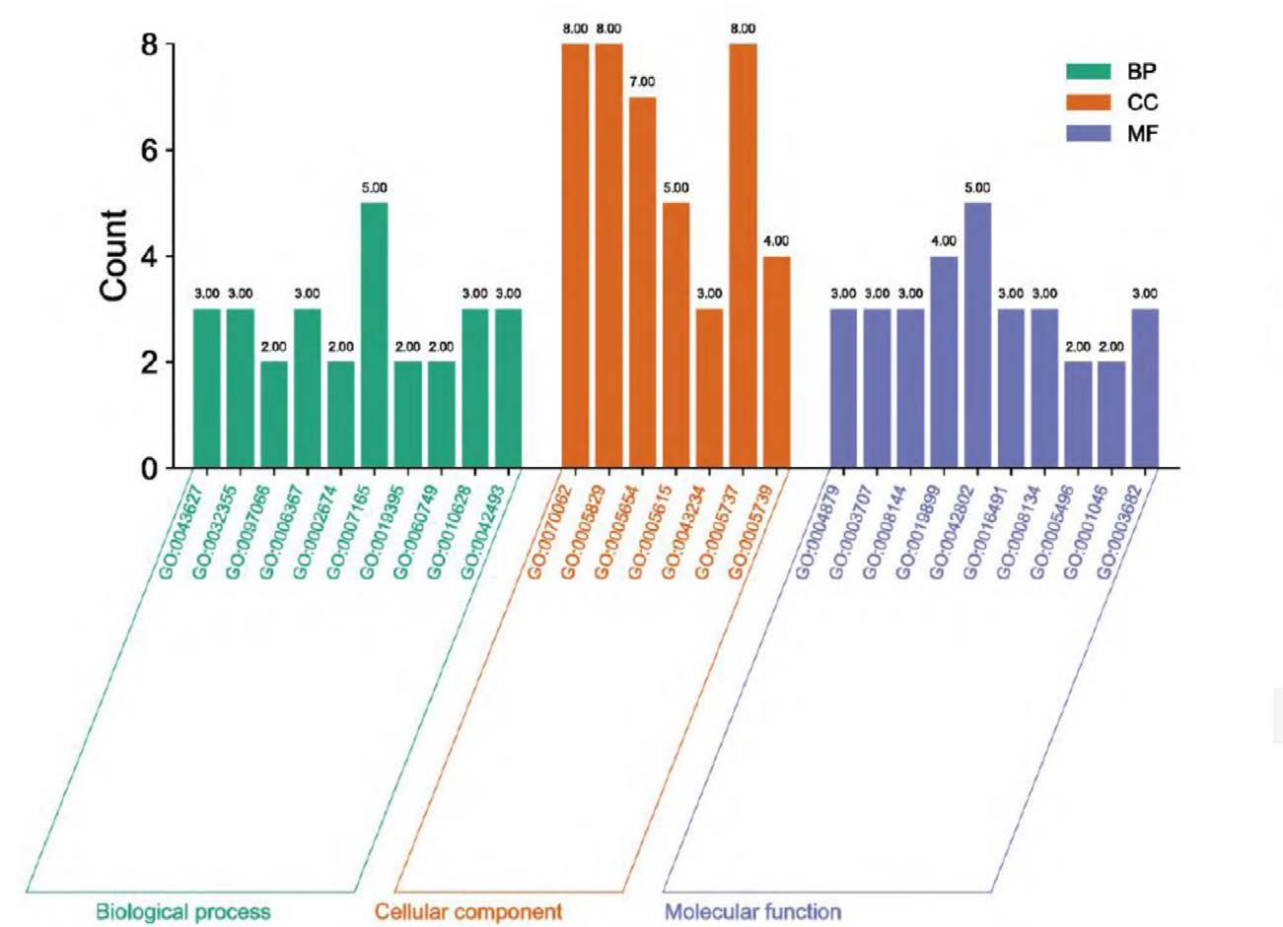
Key drug targets of the Chuanxiong Rhizoma, Radix Angelicae Biseratae and Achyranthis Bidentatae Radix combination (Note: The graph indicates the proportion of key targets in the Chuanxiong Rhizoma, Radix Angelicae Biseratae and Achyranthis Bidentatae Radix combination, and the vertical coordinate shows a decreasing trend in the proportion of key targets from the bottom to the top).

The PPI network of chemical components and drug-disease shared targets of Chuanxiong Rhizoma, Radix Angelicae Biseratae and Achyranthis Bidentatae Radix combination was built using Cytoscape software. The CytoNCA plug-in was used to complete the further topology of PPI and complete the screening of the core targets. It concluded that the top 5 nodes in terms of degree value were SOD1, CAT, AKT1, CASP3, and PTGS2. Refer to Figures 3 and 4.

### 3.5. Enrichment analysis of GO and Kyoto Gene and Gene Encyclopedia pathways

**Figure 5.**
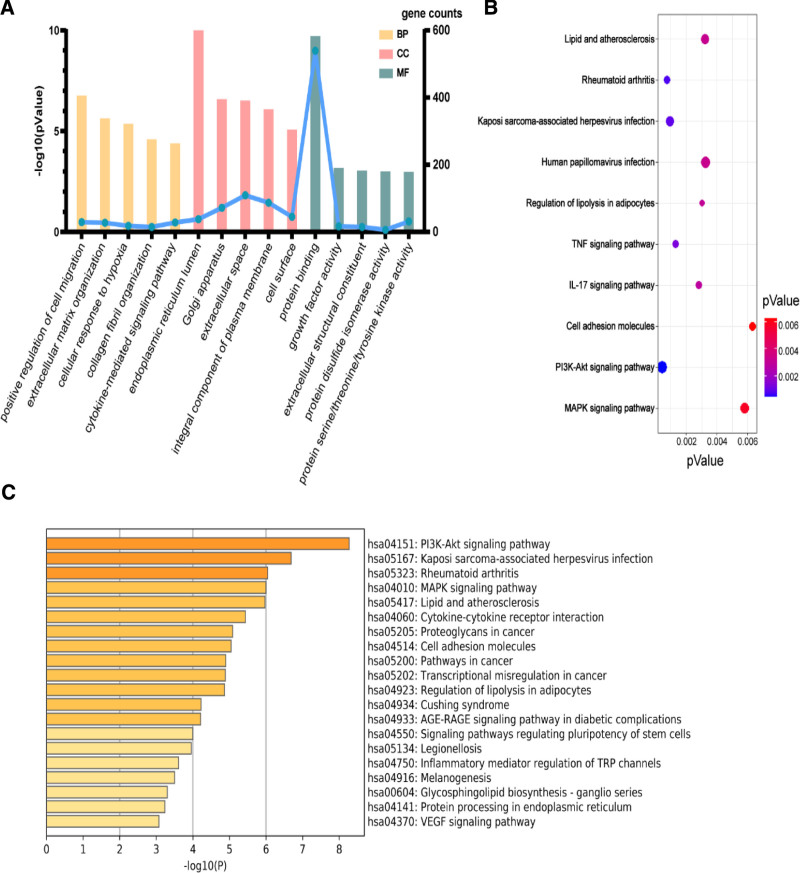
Enrichment analysis of the GO and KEGG pathways (Note: Figure A shows the number of different genes; Figure B shows the bubble map for KEGG pathway enrichment analysis; Figure C shows the picture of Genome enrichment picture of Chuanxiong Rhizoma, Radix Angelicae Biseratae and Achyranthis Bidentatae Radix combination for arthritis). GO = gene ontology, KEGG = Kyoto Gene and Gene Encyclopedia.

The GO enrichment analysis was completed using DAVID database, R4.0.2 software, and 1332 entries were obtained, including 1120 entries for biological processes, 112 entries for cellular components, and 100 entries for molecular functions. The cell component involves cell fraction, extracellular space and insoluble part. The targets in molecular function involve amine binding, drug binding, and amine receptor activity, as shown in Figure 5.

## 4. Discussion

Arthritis is a multifactorial condition that has multiple causes. It is also a fundamental lesion in the pathological process of degenerative knee osteoarthritis. Current research has made effective intervention and treatment strategies for arthritis a popular topic.^[15]^ Research conducted by Pagliai G et al^[16]^ demonstrated that the body’s immunological balance may have a direct role in the onset and progression of osteoarthritis. An inflammatory response may be sparked by an immunological imbalance brought on by a disruption in the body’s Th17/Treg cell balance. Research^[17]^ has verified that the secretion of interleukin-10 by Treg cells in patients with arthritis can ameliorate osteoarthritis. Th17 cells secrete interleukin-17, which can enhance local inflammation, promote immunity, and cause the release of other inflammatory cytokines. The research results above suggest that improving the inflammatory response and balancing the Th17/Treg cell immune response could be useful therapy strategies for arthritis. In this study, a total of 14 effective chemical components were obtained after screening, with a total of 340 corresponding targets, and 171 active ingredient targets were obtained after screening and deweighting. The protein was converted into gene names, and a total of 75 targets were obtained, which were used as candidate target sources for prescription. The study’s findings demonstrate how complex and challenging it is to identify the precise mechanism by which traditional Chinese medicine works on patients with arthritis. This is particularly true of its compound, which has the ability to target and affect multiple targets at once and can be used as a guide for treating arthritis. The study’s findings demonstrate how complex and challenging it is to identify the precise mechanism by which traditional Chinese medicine works on patients with arthritis. This is particularly true of its compound, which has the ability to target and affect multiple targets at once and can be used as a guide for treating arthritis.^[18]^

The main ingredients of Guanjietong Pian, a popular proprietary Chinese medicine used in clinical settings, are Chuanxiong Rhizoma, Radix Angelicae Biseratae and Achyranthis Bidentatae Radix. These ingredients can stimulate blood circulation, tonify the kidney and liver, and improve the course of arthritis. Previous research^[19,20]^ has demonstrated that Guanjietong Pian in conjunction with arthroscopic cleaning for arthritis patients can decrease the amount of interleukin-6 and tumor necrosis factor-α in patients with osteoarthritis of the knee and postpone the need for an artificial joint replacement. In addition, Guanjietong Pian have the ability to reduce inflammatory exudation and lessen the traumatic inflammatory response following anterior cruciate ligament restoration of the knee. In this work, 41 targets were obtained with the aid of relevant tools after Chuanxiong Rhizoma, Radix Angelicae Biseratae and Achyranthis Bidentatae Radix medication targets and arthritis targets were imported into R program; The top 5 nodes in terms of degree value were PTGS2, AKT1, CASP3, CAT, and SOD1, after the CytoNCA plug-in was utilized to finish the PPI topology; and the GO enrichment analysis yielded 1332 entries, including 1120 entries related to bioprocesses, 112 entries related to cellular composition, 100 molecular activities, etc. The Chuanxiong Rhizoma, Radix Angelicae Biseratae and Achyranthis Bidentatae Radix combination has multiple targets with arthritis, as demonstrated by the study’s results. This combination may slow down the disease’s progression by increasing the abundance of Streptococcus in the intestinal flora and raising isovaleric acid levels, which will inhibit Treg cell differentiation and affect the Th17/Treg cell immune balance.^[21]^ Analysis of the reasons: Chuanxiong Rhizoma, Radix Angelicae Biseratae and Achyranthis Bidentatae Radix pairing can improve the structure of intestinal flora, improve the immune balance of the body, and then alleviate the symptoms of osteoarthritis, which can provide new ideas and methods for the clinical treatment of osteoarthritis.^[22]^

In summary, the Chuanxiong Rhizoma, Radix Angelicae Biseratae and Achyranthis Bidentatae Radix combination can play a different role in the compatibility of other drugs and provide positive outcomes for the treatment of arthritis. The mechanism underlying this may be connected to the control of the immunological balance between Th17/Treg cells. Therefore, Chuanxiong Rhizoma, Radix Angelicae Biseratae and Achyranthis Bidentatae Radix combination can be used clinically to reduce inflammation and alleviate the symptoms of arthritis patients.

However, our research has some limitations. Although our findings provide a foundation for future research into the potential treatment of arthritis with Chuanxiong Rhizoma, Radix Angelicae Biseratae and Achyranthis Bidentatae Radix combination, the results of this study are based on data analysis and have only a certain predictive effect, which still needs to be verified by further in vitro and in vivo experiments.

## Author contributions

**Conceptualization:** Zhiqing Chen, Binshan Zhang, Zhen Li.

**Data curation:** Zhiqing Chen, Chaoyi Yin.

**Formal analysis:** Zhiqing Chen, Chaoyi Yin, Binshan Zhang.

**Methodology:** Zhiqing Chen, Zhen Li.

**Project administration:** Zhiqing Chen, Chaoyi Yin, Binshan Zhang, Zhen Li.

**Writing – original draft:** Zhiqing Chen, Chaoyi Yin.

**Writing – review & editing:** Zhiqing Chen, Binshan Zhang, Zhen Li.
